# Coordinated Expression Domains in Mammalian Genomes

**DOI:** 10.1371/journal.pone.0012158

**Published:** 2010-08-18

**Authors:** Yong H. Woo, Michael Walker, Gary A. Churchill

**Affiliations:** The Jackson Laboratory, Center for Genome Dynamics, Bar Harbor, Maine, United States of America; Dana-Farber Cancer Institute, United States of America

## Abstract

**Background:**

Gene order in eukaryotic genomes is not random. Genes showing similar expression (coexpression) patterns are often clustered along the genome. The goal of this study is to characterize coexpression clustering in mammalian genomes and to investigate the underlying mechanisms.

**Methodology/Principal Findings:**

We detect clustering of coexpressed genes across multiple scales, from neighboring genes to chromosomal domains that span tens of megabases and, in some cases, entire chromosomes. Coexpression domains may be positively or negatively correlated with other domains, within and between chromosomes. We find that long-range expression domains are associated with gene density, which in turn is related to physical organization of the chromosomes within the nucleus. We show that gene expression changes between healthy and diseased tissue samples occur in a gene density-dependent manner.

**Conclusions/Significance:**

We demonstrate that coexpression domains exist across multiple scales. We identify potential mechanisms for short-range as well as long-range coexpression domains. We provide evidence that the three-dimensional architecture of the chromosomes may underlie long-range coexpression domains. Chromosome territory reorganization may play a role in common human diseases such as Alzheimer's disease and psoriasis.

## Introduction

Gene order in eukaryotic genomes is not random. Neighboring genes are more likely to be co-expressed than distant genes. Evidence for this genome-wide phenomenon has been shown in many eukaryotes, including yeast, fruit fly, mouse, and human [1],[2],[3],[4]. Coexpression should reflect common gene function and local coexpression may be partially explained by clustering of duplicated genes. However, even after accounting for the effects of gene duplication, significant clustering of genes in the same biological pathways can still be observed [5].

Several mechanisms could potentially drive coexpression of nearby genes [3]. Neighboring genes in a divergent orientation can share a common bi-directional promoter that mediates coexpression [6]. Nearby genes may exhibit coexpression due to local chromatin configuration, often demarcated by boundary elements such as insulators [7]. Genes sharing the same chromosome territories in the nucleus may exhibit coexpression even when they are distant in the linear genome [8]. We carried out a systematic analysis of expression correlation over a range of physical scales in order to identify the factors that contribute to local and long-range patterns of coexpression. Our data are drawn from expression surveys in mouse and human and we conjecture that our findings will apply to other mammalian species.

## Results and Discussion

### Data

We assembled a large collection of microarray data, including tissue surveys, genetic mapping studies, small-molecular perturbation of cell lines, and comparisons of diseased and normal tissues, generated using several microarray platforms (Table 1; Table S1). Previous studies of coexpression have focused primarily on human tissue surveys [3]. We have also included data from mouse studies and selected studies with various types of perturbations in order to explore the generality of observed correlation patterns.

**Table 1:** A summary of gene expression data sets used in the study.

Species	Primary Perturbation Category	sample size (range)	Number of Datasets	Microarray Platforms
Human	Tissue	73	1	Affymetrix^1^
	Genetic	427–1240	3	Affymetrix^1^, Illumina^1^, Rosetta/Agilent^2^
	Chemical	2335	1	Affymetrix^1^
	Disease	23–116	5	Affymetrix^1^
Mouse	Tissue	47–61	2	Affymetrix^1^
	Genetic	120–295	3	Affymetrix^1^, Rosetta/Agilent^2^

1 = single-channel microarray platform;

2 = dual channel microarray platform.

### Coexpression in Tissue Surveys

**Figure 1:**
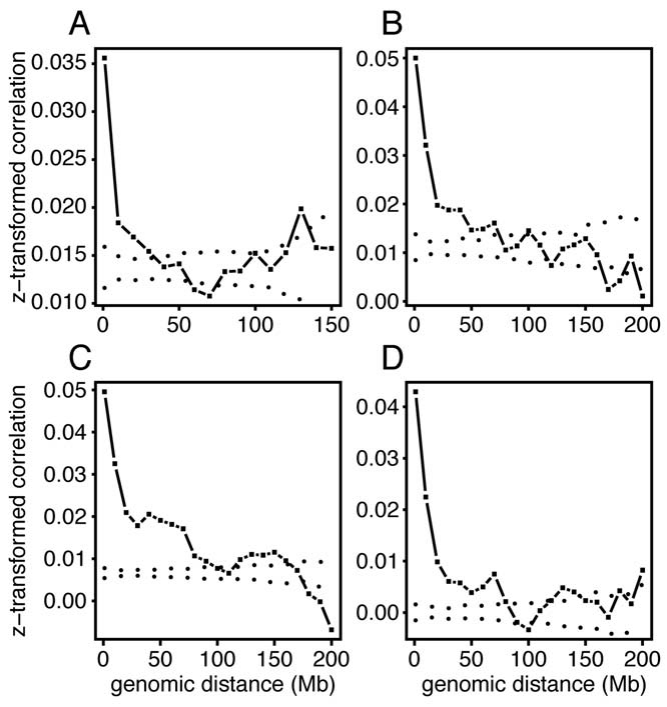
Short- and long-range coexpression in mouse and human data. Average coexpression (z-transformed Pearson's correlation coefficients) between gene pairs is shown as function of intergenic distance (base pairs). Dotted lines indicate the 95% confidence interval as determined by permutation analysis. (A) Mouse tissue expression data [4] (B) Human tissue expression data [4] (C) Lymphocyte expression data [10] (D) Small-molecule survey [11].

In two tissue surveys, one of 61 tissues in mouse and another of 73 tissues in human [4], we computed measures of coexpression for all pairs of genes over a range of intergenic distances (see Methods). We detected strong and statistically significant enrichment of coexpression among pairs of genes whose distances fall within the sub-megabase range (Figure 1A,B). We also detected weaker but still significant enrichment of coexpression among genes with distances spanning tens of megabases.

**Figure 2:**
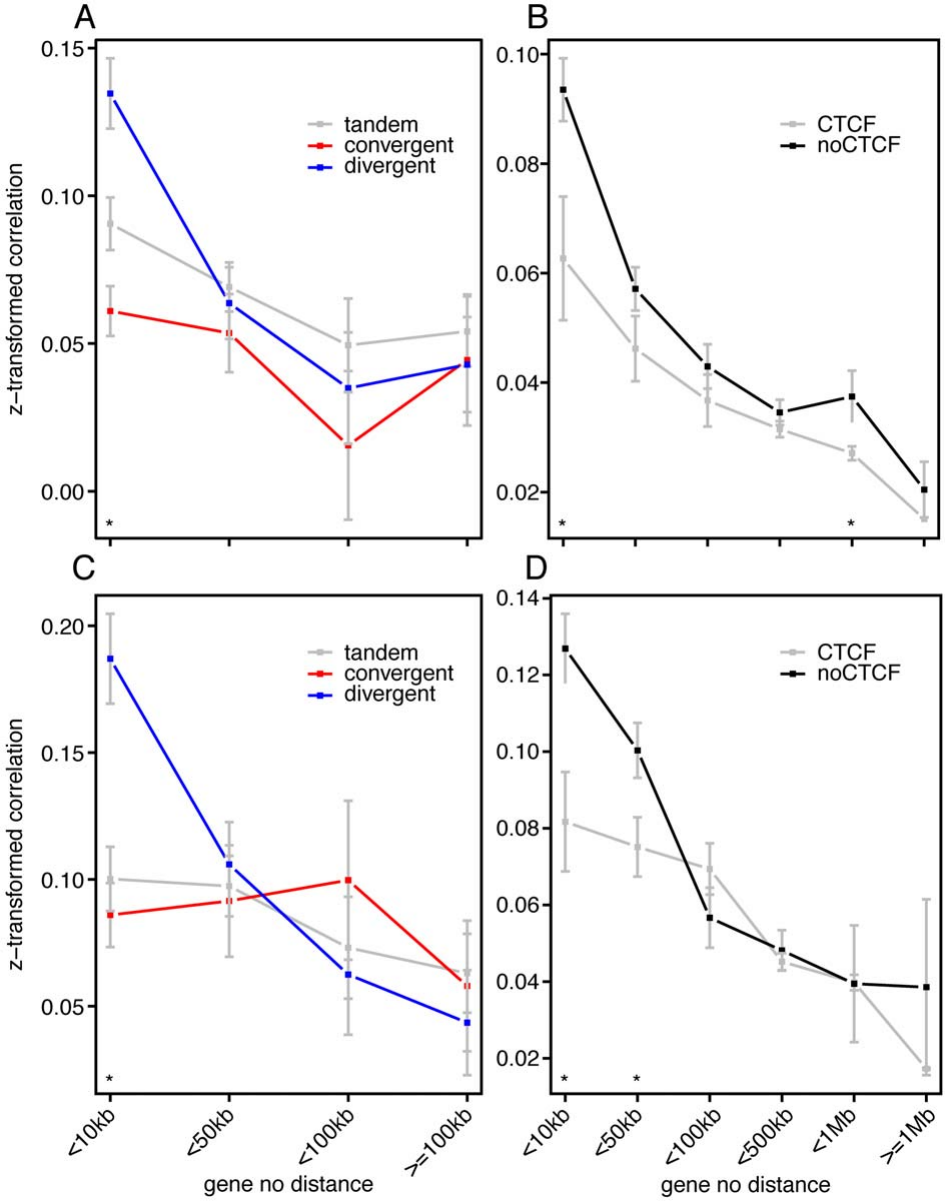
CTCF binding sites and gene orientation are determinants of coexpression. (A),(C) Average coexpression between gene pairs was plotted as a function of relative orientation and intergenic distance for adjacent genes. (B),(D) Average coexpression between gene pairs as a function of their intergenic distance in basepair and the presence or absence of intervening CTCF binding sites. Gene pairs with distance larger than 1 Mb were grouped as one. Statistical significance of differences between groups was assessed for each distance group, and displayed by asterisk (*) if p-value is less than 0.05. (A),(B) 61 mouse tissue survey (C),(D) 73 human tissue survey [4].

We tested the orientation of gene pairs as an explanation for sub-megabase range coexpression. A gene pair can be in tandem (++ or −−), divergent (−+), or convergent (+−) orientation. For adjacent gene pairs with small intergenic distances, we observed significantly higher coexpression when in divergent orientation (Figures 2A,C) (Figure S1). This is consistent with a previous study demonstrating the potential for adjacent divergent gene pairs to share a common bi-directional promoter [6].

Next we tested the effect of CTCF binding sites on coexpression. CTCF is a mammalian insulator that marks boundaries of regions under the control of enhancer elements [7]. After stratifying for intergenic distances, we detected higher coexpression in gene pairs with no known intervening CTCF site (Figures 2B,D). This effect was limited to sub-megabase scales, as the likelihood of a gene pair with no intervening CTCF site diminishes at larger scales. We repeated the analyses in an independent mouse tissue survey [9] and detected the same result (Figure S2).

### Coexpression in Genetically and Chemically Perturbed Samples

We examined lymphocyte gene expression data obtained on a sample of 1240 individuals [10], and we detected short-range coexpression clustering as seen in the tissue survey data. However, long-range coexpression clustering, from 20 Mb to more than 70 Mb, was more prominent (Figure 1C).

Genetic variation provides a powerful perturbation of gene expression. To investigate the possibility that the observed long-range coexpression signal was unique to genetic variation studies, we examined a set of samples (2335) in which a series of small molecules was applied to perturb a single cell line MCF7 [11]. We observed the same patterns of short- and long-range coexpression, indicating that these are not specific to tissue, genetic, or chemical perturbations (Figure 1D).

The long-range coexpression signatures are statistically significant but small in magnitude. A previous study has concluded that coexpression is restricted to nearby genes [12]. The disagreement with our findings may be due to the small sample sizes used in previous studies. For example, when the sample size is 10, a correlation coefficient of r = 0.63 would be statistically significant (p<0.05). The detection level for significant correlation drops to r = 0.28 when the sample size is 50. For a sample of size 1240 the smallest significant correlations would be r = 0.056. Large sample sizes facilitate discovery of subtle, long-range coexpression clustering.

### Mosaic structure of long-range coexpression

**Figure 3:**
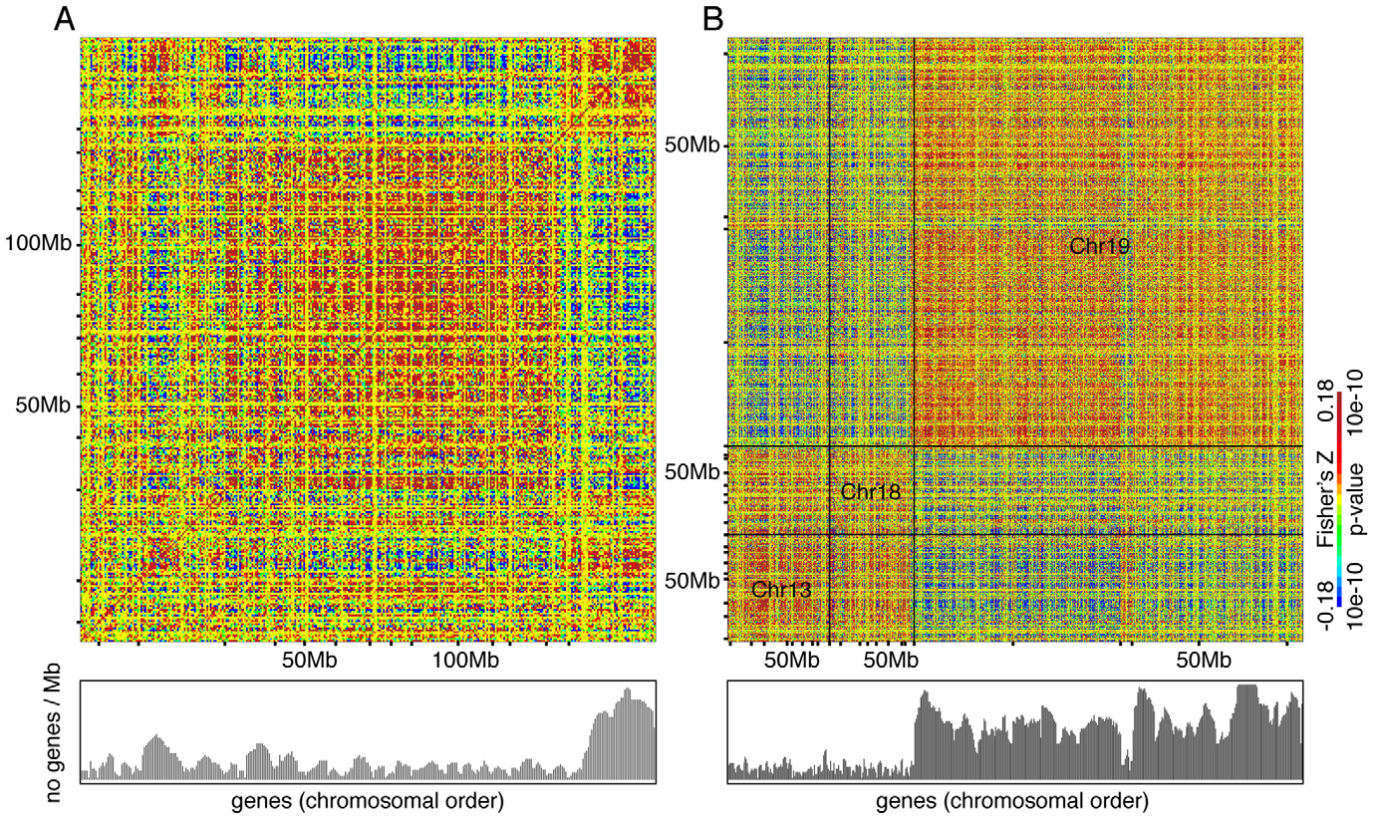
Evidence for coexpression domains. Z-transformed Pearson's correlation coefficients are displayed as a heat-map. The magnitude of the correlation coefficients is displayed using a color scale, truncated at the range displayed in the legend. Gene density, defined as the number of protein-coding genes in a 1 Mb window centered on each gene, in the lower panel; they were truncated at 50 genes/Mb. (A) Human chromosome 8 (B) Human chromosomes 13, 18, and 19. Chromosomes are demarcated by dotted lines.

Domain correlations appear to be pervasive and are distributed in a genome-wide mosaic pattern (Figure 3A). We observed identical patterns of coexpression domains in other human datasets (Figures S3A,B). Furthermore, when we remapped mouse genes to their orthologous positions on human chromosomes, we found that the mouse coexpression domains were concordant with the human domains (Figures S3C,D). Concordance across two distantly related mammalian species suggests that the mosaic structure of coexpression domains is broadly conserved across mammals.

Could these correlations be spurious technical, microarray artifacts, for example by base composition of the probe? Given that these data are generated by microarray platforms diverse in terms of the array manufacturing method, the hybridization method, or probe designs, it is not likely that platform-specific biases influence these results (Table 1). Furthermore, any fixed feature of probe performance such as GC content that affect intensity cannot explain a correlation, which depends on coordinated variation in intensity.

Human genomes contain large genomic regions characterized by high and low gene expression that are distinct in terms of gene-density, GC-nucleotide content, intron size, and replication timing [13], [14]. However, fixed patterns of high and low expression will not result in correlation, which can only arise in the presence of variation in expression levels. Therefore the presence of regions of high and low expression is not sufficient to explain domains of co-expressed genes. The patterns observed here can be explained by coordinated changes in gene expression across large genomic domains.

### Evidence for genome-wide coordination of coexpression domains

**Figure 4:**
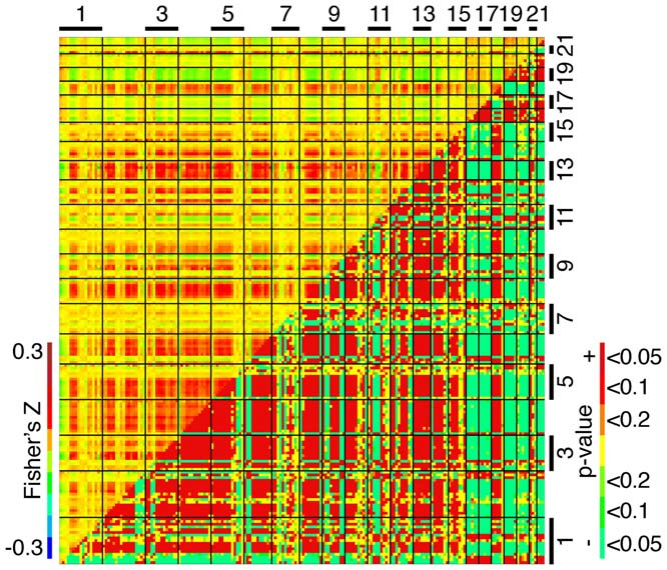
Genome-wide correlations between coexpression domains. Correlations between 30 Mb coexpression domains (15 Mb overlapping) on a genome-wide scale. The upper triangle represent mean z-transformed correlation coefficients; the lower triangle statistical significance determined by permutation analysis (100 permutations), with the threshold indicated by the color scale on the right side legend.

Domains of coexpression can be positively or negatively correlated. To detect correlations between domains from the same chromosome as well as from different chromosomes on a genome-wide basis, we analyzed correlations between 30 Mb windows equally spaced across the genome with 15 Mb overlap. We assessed statistical significance using genome-wide permutation analysis (see Methods). Throughout the genome, we found evidence of extensive correlations between domains within as well as across chromosomes (Figure 4) (Figure S5). As an example of intra-chromosomal correlations, the domain spanning 30 to 130 Mb window on human chromosome 8 is negatively correlated with the domain spanning 130 Mb to distal end on the same chromosome (Figure 3A). As an example of inter-chromosomal correlations, domains on chromosomes 13 and 18 are positively correlated with one another, but they are negatively correlated with a domain spanning chromosome 19 (Figure 3B). We detected similar genome-wide patterns in other human datasets (Figure S4A,B) (Figure S5A). When mouse genes were remapped to orthologous positions on the human genome, we detected the same correlation pattern between many coexpression domains, indicating evolutionary conservation of relationship between coexpression domains (Figure S5B). Our results suggest pervasive, genome-wide interaction between co-expression domains.

### Density-dependent correlations

**Figure 5:**
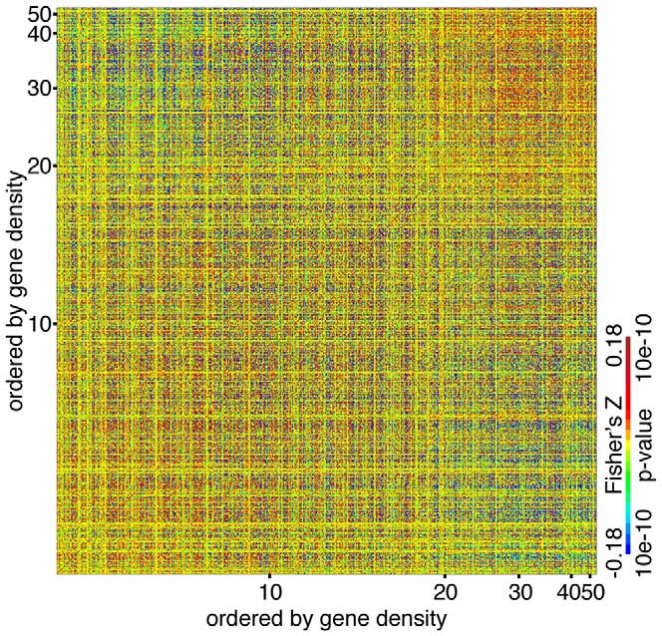
Genome-wide association between coexpression and gene density. Correlation heatmap similar to Figure 3, except that the horizontal and vertical positions are ordered by gene density, not by chromosomal positions. Every 10th genes were sampled for visualization purpose.

In the human genome, gene-rich regions are found more often in large chromosomes, whereas gene-poor regions are found in small chromosomes. We generally detected positive correlations when both domains come from large chromosomes or small chromosomes, but negative correlation between a domain of a small chromosome and a domain of a large chromosome, suggesting that correlations between coexpression domains depend on gene density (Figure 4). To test this on a genome-wide basis, we generated a correlation heat-map ordered by gene density instead of genomic position. Genes with similar gene density were positively correlated with each other, while those from low density regions were negatively correlated with those from high density regions (Figure 5). Permutation analysis confirmed that the observed correlation between gene density and gene expression similarity was statistically significant (1 out of 500 permutations, p = 0.002). Association of gene density with coordinated changes in expression between chromosomal domains is a pervasive and genome-wide phenomenon.

### Chromosomal territory and coexpression domains

**Figure 6:**
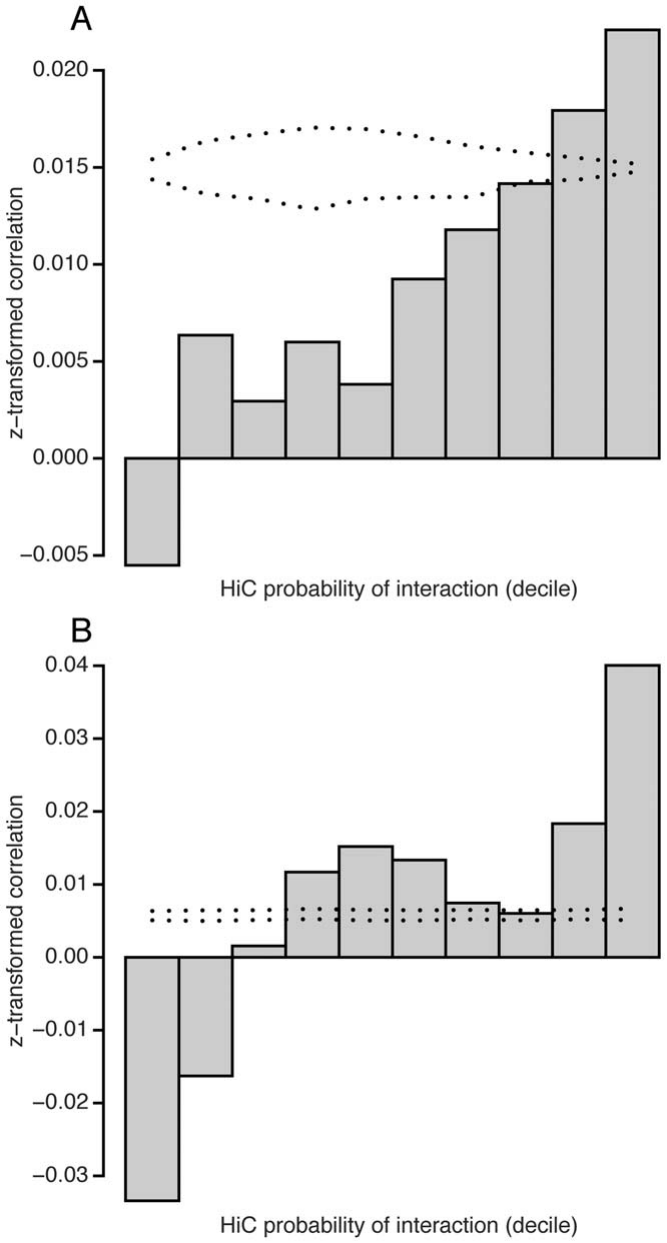
Genome-wide association between coexpression and spatial proximity. Barchart showing average z-transformed correlation coefficients for 10 groups according to the spatial proximity (“probability of interaction”) between the two genes. Dotted lines 95% interval calculated from permutation analysis. (A) Intra-chromosomal gene pairs (B) Inter-chromosomal gene pairs.

What is the underlying mechanism of gene density-dependent correlated gene expression? We investigated whether the density-dependent correlations were related to organization of chromosome territories in the three-dimensional nucleus. Despite growing evidence that chromosome territory organization is involved in regulating gene expression, low-throughput capacity of traditional cytogenetic assays limited the ability to study it on a genome-wide basis [8]. A recent study developed a massively parallel sequencing-based method to detect genomic loci that are spatially nearby in the three-dimensional nucleus, and generated spatial proximity information across the whole human genome at 1 Mb resolution [15]. Using the data, we tested for association between spatial proximity and coexpression for all possible gene pairs. The analysis revealed that spatially nearby gene pairs are more likely to be be coexpressed than spatially distant gene pairs (Figure 6). This was true for both intra-chromosomal and inter-chromosomal gene pairs, indicating that the trend is not due to proximity in the linear genome (Figures 6A,B, respectively). Consistent with this, when we visually compared coexpression and spatial proximitiy domains across chromosomes, the two overlapped often (for example human chromosome 8; Figure S6). This suggests that the relationship between correlated expression and spatial proximity in the nucleus are pervasive and genome-wide.

The genome-wide spatial proximity data we used for our study represent a significant advance, providing for the first time genome-wide, high-resolution view of chromosome organization in the nucleus [15]. When we quantified density-dependent coexpression from spatial proximity-dependent coexpression by analysis of variance, the extent of coexpression explained by gene density similarity was greater than that by spatial proximity (Table S2, S3). Also, genomic regions of coexpressed genes were often close in the three-dimensional nucleus, but the agreement was not prefect (Figure S6). These suggest that there are factors other than spatial proximity which contribute to genome-wide coordinated expression changes.

Our understanding of how gene density-dependent coexpression relates to organization of three-dimensional chromosome territories could benefit from advances in the following areas. First, the genome-wide chromosome territory structure was determined on a single cell type under a constant condition, whereas genome-wide correlation reflects dynamic changes that may be related to variation in the organization of chromosome territories across a wide variety of conditions examined here. More chromosome proximity data on a wide range of cell types and perturbations could improve our understanding of how chromosome territories relate to gene expression. Second, the study focused on spatial proximity between genomic loci, but there are other important components of chromosome territory structure. For example, combining information on spatial proximity between genomic loci with information on spatial proximity to the nuclear lamina could yield for a refined picture of chromosome territory structure [16],[17]. Third, gene density may be associated with differences in biological function, and how the mosaic structure of gene-rich and gene-poor domains in the mammalian genome is inter-related with functional organization, three-dimensional chromosome organization, and gene expression regulation would provide a comprehensive view of the role of genome organization in cellular processes.

### Density-dependent gene expression changes correlate with disease

To investigate the possible relationship between coexpression domains and phenotypes important for human health, we tested whether transcriptional changes that occur during disease progression drive correlations with the same domain structure.

**Figure 7:**
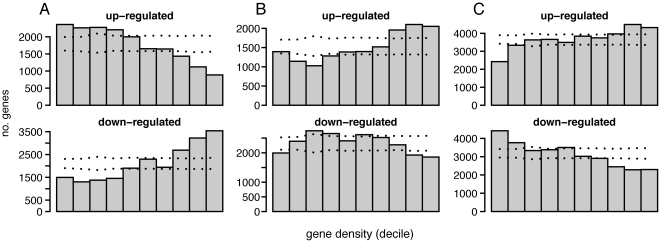
Density-dependent gene expression changes in diseased samples. Barchart showing the number of genes up-regulated (gray bar) or down-regulated (white bar) in diseased tissues compared to control (p<0.05). Genes were divided into 10 groups of equal size, according to the gene density. Dotted lines indicate 95% interval calculated from genome-wide permutation, shuffling regional gene density associated with each gene. (A) Entorhinal cortex [18] (B) Hippocampus [18] (C) Skin lesions and matched normal sample in psoriasis patients [20].

We chose to look at Alzheimer's disease, because of its prevalence and impact on human health, and due to the availability of a high quality collection of gene expression profiles [18]. We compared the gene expression profiles between normal and diseased samples in the entorhinal cortex and hippocampus, key tissues implicated in Alzheimer's disease. Gene expression in entorhinal cortex of Alzheimer's patients were marked by significant up- and down-regulated genes in low and high gene-density regions, respectively (Figure 7A) (Figure S7). Interestingly, we detected an opposite trend in hippocampus tissues, suggesting that domain-wide expression changes during disease progression can be tissue-specific (Figure 7B). When we repeated the analysis in an independently generated hippocampus data, we detected the same trend (Figure S8A) [19].

We investigated psoriasis, because high-quality gene expression data were available for both normal and disease samples from the same patient, providing an internal control for genetic variability [20]. We detected significant up- and down-regulation of genes in high-density and low-density regions, respectively (Figure 7C). We detected the same trend from an independent psoriasis gene expression study, indicating that density-dependent, genome-wide gene expression changes is a salient feature of psoriasis progression (Figure S8B) [21].

Evidence for alterations in chromosome territories during disease progression have been documented in diseases such as immunodeficiency centromeric instability facial abnormalities and cancer [22],[23],[24]. However, the generality of this phenomenon is largely unknown. These results indicate that many diseases show signs of density-dependent, genomic location-dependent gene expression changes, suggestive of underlying changes in chromosome territory positions within the nucleus. The relationship between three-dimensional chromosome organization and gene expression alterations underlying disease progression warrants further investigations.

In conclusion, we have investigated a variety of factors that can potentially explain short (<1 Mb) and long-range (>10 Mb) clustering of co-expressed genes in the mammalian genome. Short-range correlation in gene expression is present even after accounting for local gene duplications. It is partially explained by gene orientation and the presence of insulator elements that allow pairs or groups of genes to synchronously vary in their expression. Long-range clustering occurs in mosaic coexpression domains across the genome. These domains are not independent but rather show extensive correlation with other domains. Correlations between domains are conserved between mouse and human and are associated with variation in gene-density and positioning of chromosome territories in the nucleus. We provide evidence for a potential association between coordinated genome-wide changes in gene expression and disease status, including Alzheimer's disease. We further propose that correlations between long-range coexpression domains reflect rearrangements of chromosome territories and that this remodeling of chromosomes may play a role in disease progression. A comparison of the domain structures reported here with a recent report [15] describing genome-wide organization of chromosome territories yields some striking similarities. The agreement was not perfect, underscoring the need to examine chromosome territory structure under a variety of conditions in order to determine if more than one stable state exists. The impact of chromosome territory organization on gene expression and the dynamic interaction of these genome-wide processes upon cellular perturbation remains a subject for future investigations.

## Materials and Methods

### Microarray Data

Gene expression microarray data were collected for both mouse and human. See Table 1 for summary and Table S1 for details. Reference S1 lists literature sources for Table S1. Affymetrix data were processed using the default Robust Multiarray Algorithm (*rma*) in the R/affy package [25], using customized probe CDF libraries (version 11), which remapped all Affymetrix probes to NCBI Entrez genes [26]. Replicate arrays for tissue survey data were averaged. For Rosetta microarray platforms, we used mapping between probes and NCBI Entrez genes available at Gene Expression Omnibus (GEO) database. For Illumina platform, we used mapping using nuID database [27]. When multiple probes are mapped to the same Entrez gene, we selected the probe (or probeset) with the largest variance across samples. The small-molecule perturbation survey comprised 4508 experiments (microarrays) conducted in three cell lines: MCF7, HL60, and PC3 [11]. We focused on MCF7 only (2335 experiments).

### Definitions of Genomic Parameters

#### CTCF-binding Site

We obtained CTCF-binding coordinates from Insulator Database (http://insulatordb.utmem.edu) [28]. We used computationally predicted sites only.

#### Intergenic Distance

Genomic locations of NCBI Entrez genes were obtained in base-pair coordinates from NCBI map viewers for mouse (mm8) and humans (hg18). Intergenic distance between two genes are defined as the closest distance between them, using 5′ or 3′ end. Intergenic distances were then binned into 10 Mb intervals. Genes less than 10 Mb apart were grouped into <1 Mb and 1 Mb<x<10 Mb groups.

#### Gene Order Distance

Gene order distance was calculated based on the number of intervening genes between the two genes. Adjacent genes, having no intervening genes between the two genes of a pair, was assigned 1.

#### Gene Density

Gene density was defined as the number of protein-coding, NCBI Entrez genes in 11 Mb intervals across the genome [29].

### Removal of Duplicates

Duplicated genes are often physically clustered in the genome [3]. To avoid potential confounding of coexpressed genes, duplicated genes were removed by a novel algorithm that clusters genes together according to their annotations and genomic locations as determined by the hypergeometric distribution (MW and KP, manuscript in preparation). We applied a strict expectation threshold of e = 0.01. Random simulations show that this approach eliminated cases of false positives from our dataset. The following annotations were obtained from from Ensembl database: SCOP superfamily, Interpro domains, protein families, and gene paralogs. The algorithm was applied to each annotation system, and the resulting clusters were merged. Gaps, i.e. intervening genes, were allowed when detecting clusters, with the optimal gap size limit determined by repeating the analysis with increasing the gap size until the clustering was no longer improved.

### Three-Dimensional Spatial Proximity Data

A recent study developed a novel method combining proximity-based coupling and massively parallel sequencing technology for detecting genomic loci that are spatially nearby in the three-dimensional nucleus [15]. The study generated spatial proximity data between genomic loci at 1 Mb resolution across the whole genome for immortalized lymphoblastoid cell line GM06690 and leukemic cell line K562. We focused on GM06690 since the leukemic cells harbor cytogenetic abnormalities. We converted the Pearsons' correlation matrix into gene-centric pairwise information based on the genomic location of a gene's mid point. Spatial proximity between gene pairs whose midpoints fall in to the same 1 Mb grid were assigned a missing value.

### Calculation of Coexpression

All analyses were conducted in R environment (http://www.r-project.org). For each pair of genes, Pearson correlation coefficient between expression levels (log-transformed) of the two genes was calculated, and were Fisher's z-transformed (hyperbolic inverse tangent). Statistical significance of coexpression was assessed based on the approximation that Fisher's z-transformed correlation coefficients are normally distributed with standard error of (N-3)^−1/2^ [30]. Because the small-molecule perturbation survey [11] were conducted in batches, correlations were first calculated for each batch and were averaged across the batches, and was mean-scaled.

### Coexpression Heatmaps

A heatmap of matrix comparing pair-wise correlations was generated by ordering genes by their chromosomal orders, i.e. proximal to distal end of a chromosomes. Heatmaps generated during the study can be found at a supplementary website (http://cgd.jax.org/datasets/expression/coordinated.shtml).

### Statistical tests

#### Genomic Distance-Dependent Coexpression

To characterize distribution of coexpression as a function of intergenic distances, coexpressions, z-transformed correlation coefficients, were averaged for each intergenic distance category. To determine range of values expected by chance, we performed permutation analysis as follows. While keeping the expression value associated with each gene, we shuffled the genomic locations assigned to each gene, and averaged coexpression values for each distance category. The process was repeated 100 times, and calculated a 95% interval to represent expected range of coexpression values for each distance group.

#### Density-Dependent Coexpression

Gene density-dependent coexpression was assessed by a Pearson's correlation coefficient between coexpression and gene density difference, defined as absolute difference in log-transformed gene density. Permutation analysis was performed to assess statistical significance. We shuffled gene orders across the genome, effectively assigning a sampled gene density value while keeping the same expression value for each gene, and calculated a correlation coefficient between the coexpression and the gene density difference. The process was repeated 500 times, and statistical significance was assessed based on distribution of the permuted statistics.

#### Spatial Proximity-Dependent Coexpression

The association between coexpression and spatial proximity was analyzed similarly as density-dependent coexpression. Because the spatial data was more sparse for inter-chromosomal pairs, we analyzed intra-chromosomal gene pairs separately from inter-chromosomal pairs. We removed gene pairs whose intergenic distances are less than 10 Mb to avoid potential complications, but inclusion did not change the conclusion (data not shown). Permutation analysis was conducted by shuffling correlation coefficients 500 times (i.e. unrestricted shuffling). The analysis was repeated for inter-chromosomal and intra-chromosomal pairs, separately.

#### Comparing Gene Density and Spatial Proximity-dependent Coexpression

We performed analysis of variance (ANOVA) to delineate the extent of coexpression explained by gene density from that by spatial proximity. To quantify the contribution of one factor while accounting for the contribution of the other, we calculated type III sums of squares. The analysis was run separately for inter-chromosomal and intra-chromosomal pairs.

### Gene expression analysis of normal and diseased tissues

We obtained publicly available human gene expression profiles (Table 1; Table S1). When analyzing Alzheimer's disease data set, we detected three overt outliers detected by hiearchical clustering analysis, and remove those samples from analysis (Figure S7). Analysis including the outliers did not change our result (data not shown). We used t-test to identify differentially expressed genes between normal and diseased tissue. For psoriasis data sets, we used paired t-test as both normal and diseased sample came from the same patient.

## Supporting Information

Figure S1 Gene orientation are determinants of coexpression for adjacent genes.(A),(B) Average coexpression between gene pairs was plotted as a function of relative orientation and gene order distance. Gene pairs with gene order distance >5 (i.e. number of intervening genes are >4) were grouped as one. (A) 61 mouse tissue survey. (B) 73 human tissue survey as in Figure 2.
(0.24 MB PDF)

Figure S2 CTCF binding sites and gene orientation are determinants of coexpression. Repeat of the analysis in Figure 2 with another mouse tissue survey [9] (A) Average coexpression between gene pairs was plotted as a function of relative orientation and gene order distance. Gene pairs with gene order distance >5 (i.e. number of intervening genes are >4) were grouped as one. (B) Average coexpression between gene pairs was plotted as a function of relative orientation and intergenic distance for adjacent genes. (C) Average coexpression between gene pairs as a function of their intergenic distance in basepair and the presence or absence of intervening CTCF binding sites. Gene pairs with distance larger than 1 Mb were grouped as one. Statistical significance of differences between groups was assessed for each distance group, and displayed by asterisk (*) if p-value is less than 0.05.
(0.08 MB PDF)

Figure S3 Heatmap of correlation matrix on human chromosome 8. See Figure 3A for legends, and Table S1 for details of each data source. (A) Adipose tissues from 702 human populations (B) Liver samples from 427 human populations (C) Liver samples from 311 mouse intercross populations (D) Liver samples from 120 mouse intercross populations. Correlation heatmaps for (C) and (D) were generated by reordering mouse genes by their human ortholog locations in human chromosome 8.
(4.25 MB PDF)

Figure S4 Heatmap of correlation matrix on human chromosome 13, 18, and 19. (A)–(D) corresponds to the datasets in Figure S3(A)–(D). See Figure 3B for legends.
(5.39 MB PDF)

Figure S5 Genome-wide correlation matrix at 30 Mb resolution. See Figure 4 for legend. (A) Adipose Gene Expression from 702 human populations (Table S1). (B) Mouse co-expression,averaged across the mouse expression profiles (Table S1), using the sample size as weight. Correlation heatmaps were generated after reordering mouse genes by their human ortholog locations in the human genome.
(2.54 MB PDF)

Figure S6 Correlation heatmap of spatial proximity data for human chromosome 8. Spatial proximity information was obtained from Lieberman-Aiden et al. 2009 [15]. The corrrelation ranges from −1 to +1, and greater values indicate greater probability of contact between two genomic domains. Genes whose midpoints falling into the same 1 Mb window are indicated as white.
(0.35 MB PDF)

Figure S7 Alzheimer entorhinal cortex gene expression profile. A: Hierarchical clustering of the samples in Alzheimer entorhinal cortex gene expression study [18]. Outliers (boxed blue) were boxed. B: Heatmap showing relative gene expression profiles in diseased (top 10 rows) and normal samples (bottom 13 rows) across genes in chromosome 8 (horizontal,proximal to distal). Magenta and cyan indicate high and low expression. The outliers removed are indicated indicated by arrows. Expression level for each gene were scaled to have a mean of 0, and truncated at −1 and +1 for visualization.
(0.08 MB PDF)

Figure S8 Density-dependent Gene Expression Changes in Diseased Tissues. See Figure 7 for legends. (A) an independent study on hippocampus expression in Alzheimer's disease, corresponding to Figure 7B [19]. (B) an independent study on skin expression in psoriasis patient, corresponding to Figure 7C [21].
(0.05 MB PDF)

Table S1 Detailed list of gene expression datasets used in the study.
(0.03 MB DOC)

Table S2 Type III Analysis of variance to dissect density-dependent and spatial proximity-dependent coexpression among intrachromsomal pairs.
(0.01 MB DOC)

Table S3 Type III Analysis of variance to dissect density-dependent and spatial proximity-dependent coexpression among intrachromsomal pairs.
(0.01 MB DOC)

Reference S1 (0.02 MB DOC)
