# Supplementary figures and images for: Coordinated Expression Domains in Mammalian Genomes
Source: PLoS One. 2010 Aug 18;5(8):e12158. doi: 10.1371/journal.pone.0012158 (PMC2923606; doi:10.1371/journal.pone.0012158)

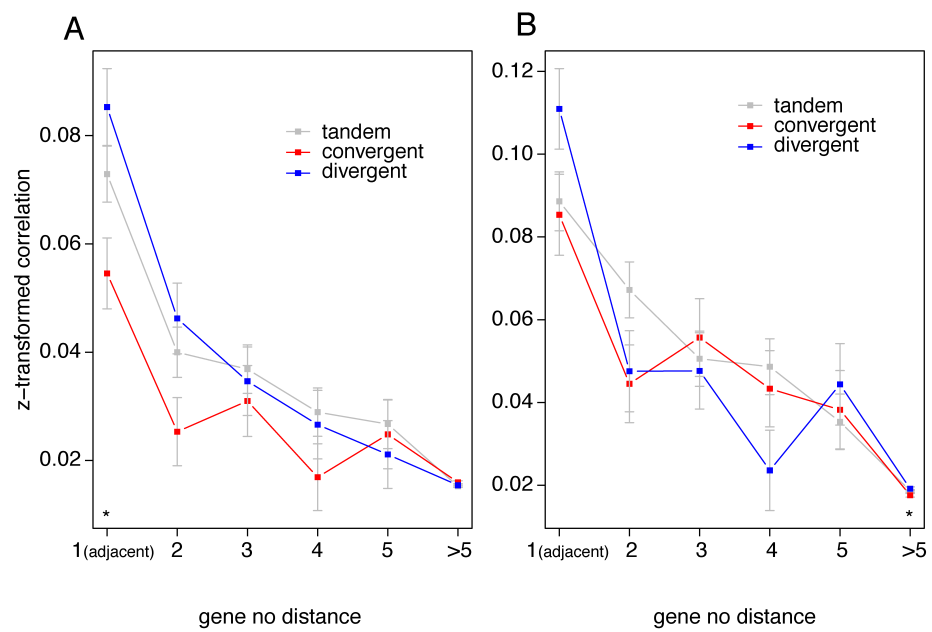

Supplement: Figure S1 — Gene orientation are determinants of coexpression for adjacent genes.(A),(B) Average coexpression between gene pairs was plotted as a function of relative orientation and gene order distance. Gene pairs with gene order distance >5 (i.e. number of intervening genes are >4) were grouped as one. (A) 61 mouse tissue survey. (B) 73 human tissue survey as in Figure 2. (0.24 MB PDF) [file pone.0012158.s001.pdf]

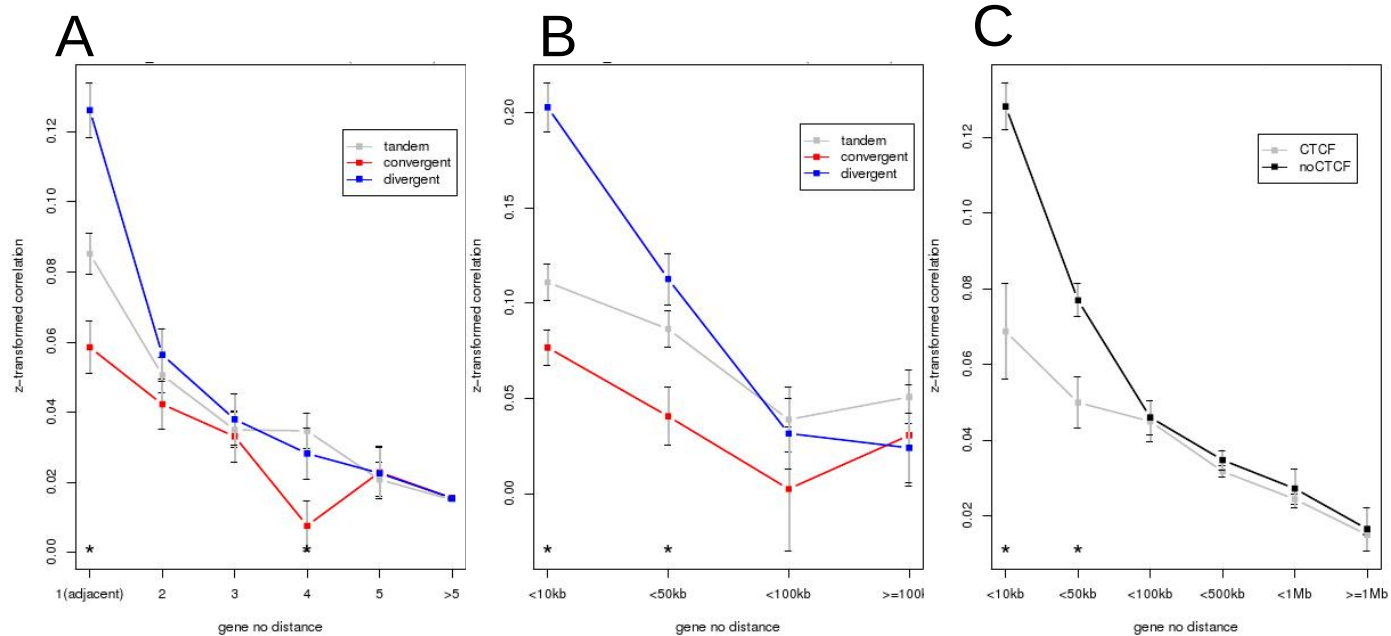

Supplement: Figure S2 — CTCF binding sites and gene orientation are determinants of coexpression. Repeat of the analysis in Figure 2 with another mouse tissue survey [9] (A) Average coexpression between gene pairs was plotted as a function of relative orientation and gene order distance. Gene pairs with gene order distance >5 (i.e. number of intervening genes are >4) were grouped as one. (B) Average coexpression between gene pairs was plotted as a function of relative orientation and intergenic distance for adjacent genes. (C) Average coexpression between gene pairs as a function of their intergenic distance in basepair and the presence or absence of intervening CTCF binding sites. Gene pairs with distance larger than 1 Mb were grouped as one. Statistical significance of differences between groups was assessed for each distance group, and displayed by asterisk (*) if p-value is less than 0.05. (0.08 MB PDF) [file pone.0012158.s002.pdf]

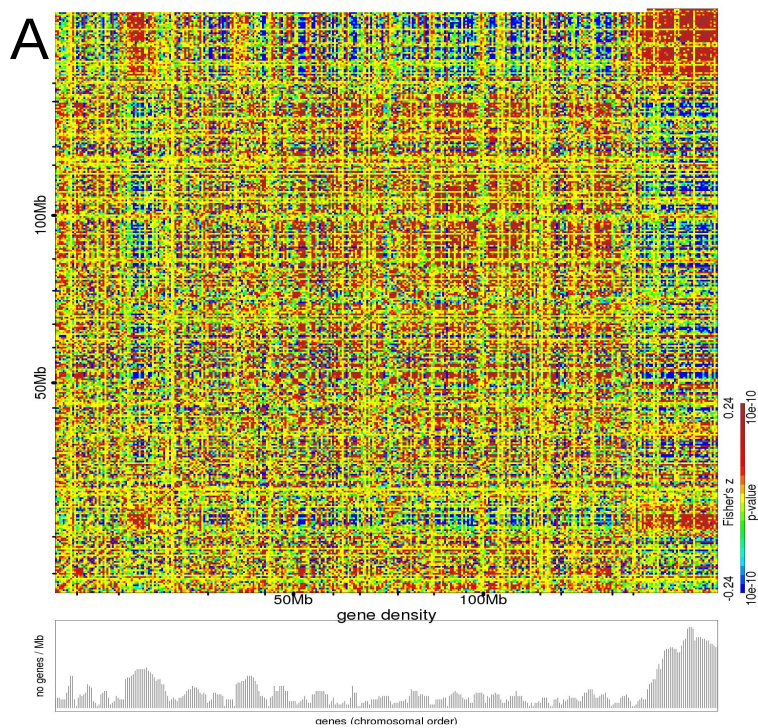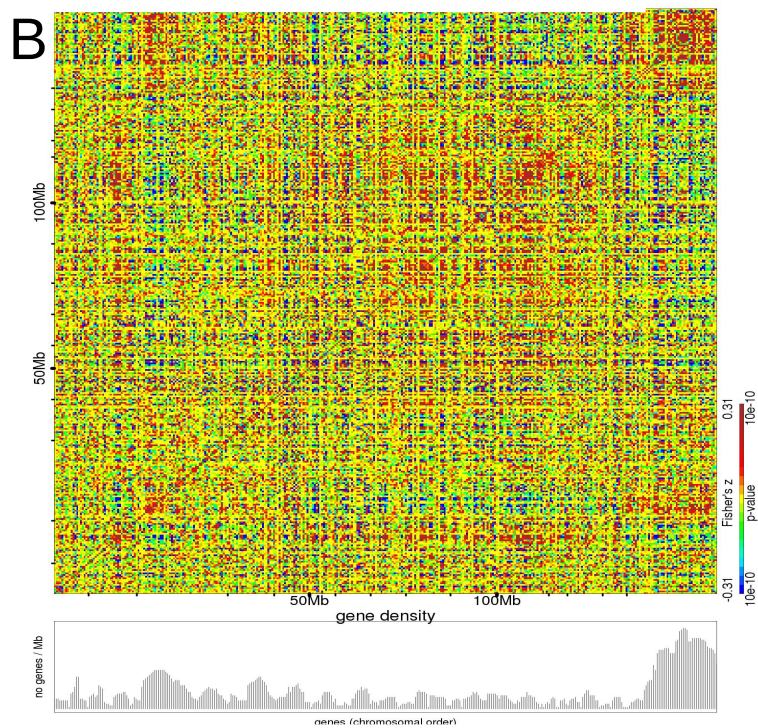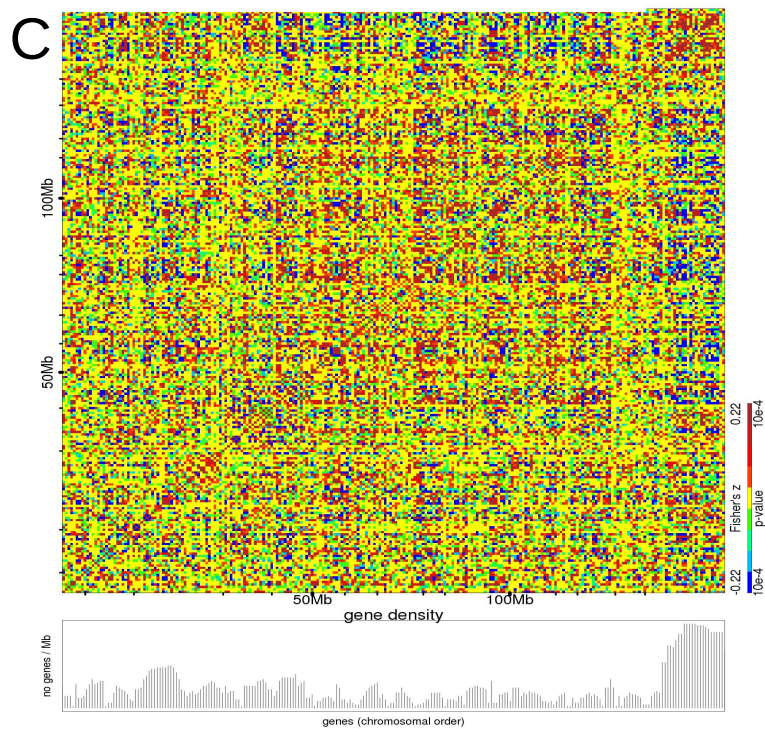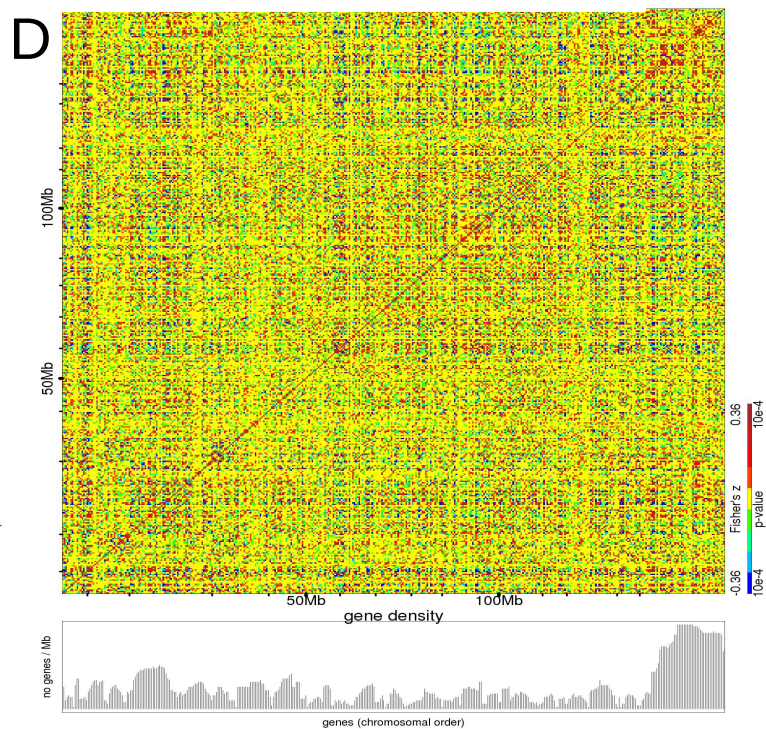

Supplement: Figure S3 — Heatmap of correlation matrix on human chromosome 8. See Figure 3A for legends, and Table S1 for details of each data source. (A) Adipose tissues from 702 human populations (B) Liver samples from 427 human populations (C) Liver samples from 311 mouse intercross populations (D) Liver samples from 120 mouse intercross populations. Correlation heatmaps for (C) and (D) were generated by reordering mouse genes by their human ortholog locations in human chromosome 8. (4.25 MB PDF) [file pone.0012158.s003.pdf]

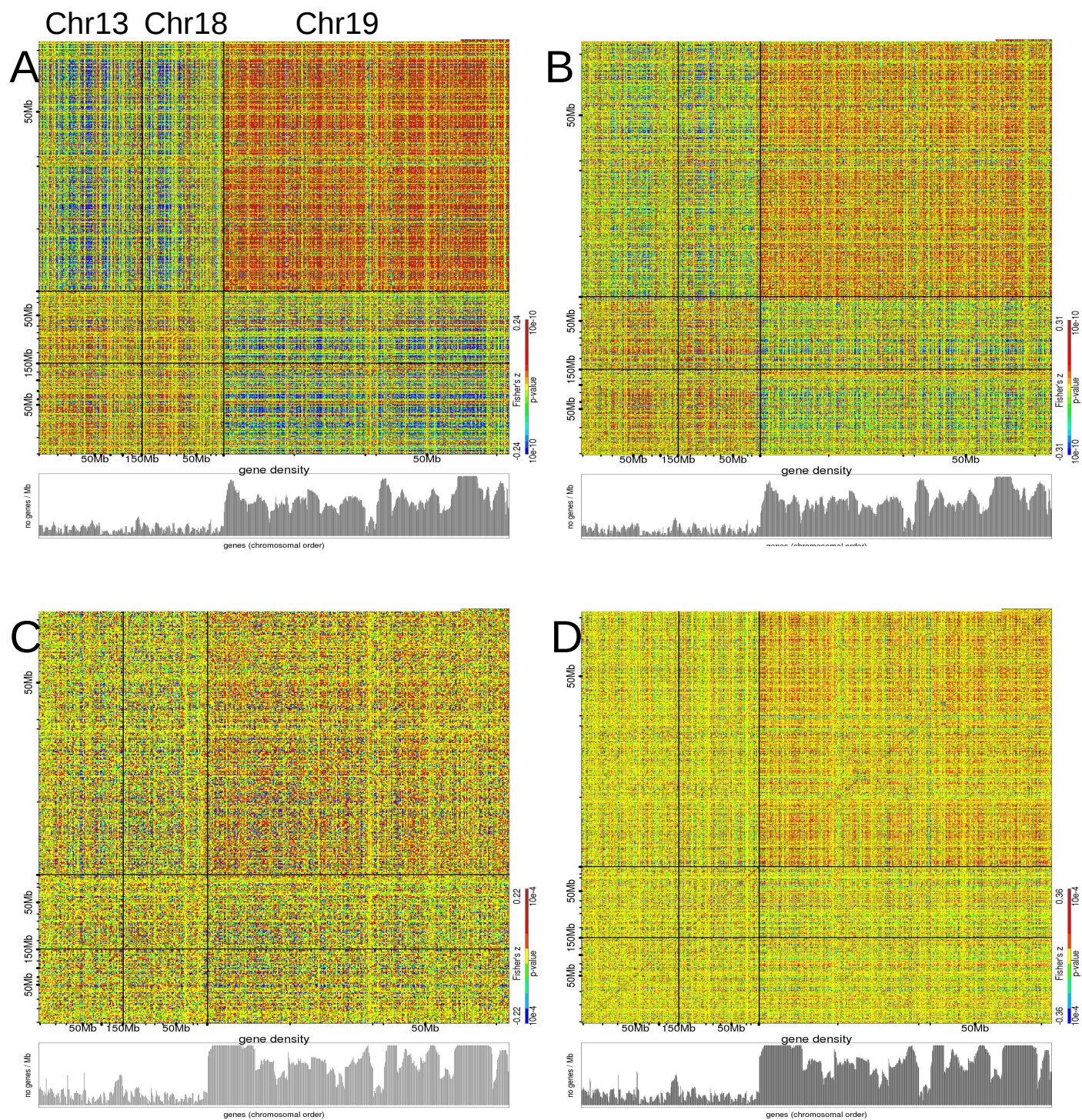

Supplement: Figure S4 — Heatmap of correlation matrix on human chromosome 13, 18, and 19. (A)–(D) corresponds to the datasets in Figure S3(A)–(D). See Figure 3B for legends. (5.39 MB PDF) [file pone.0012158.s004.pdf]

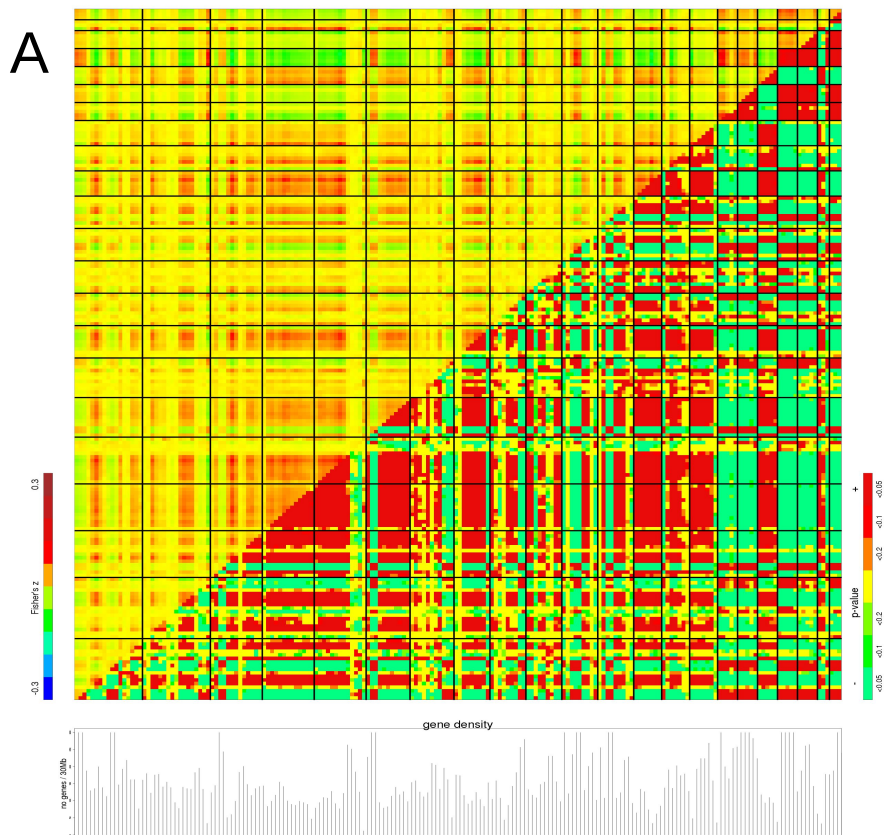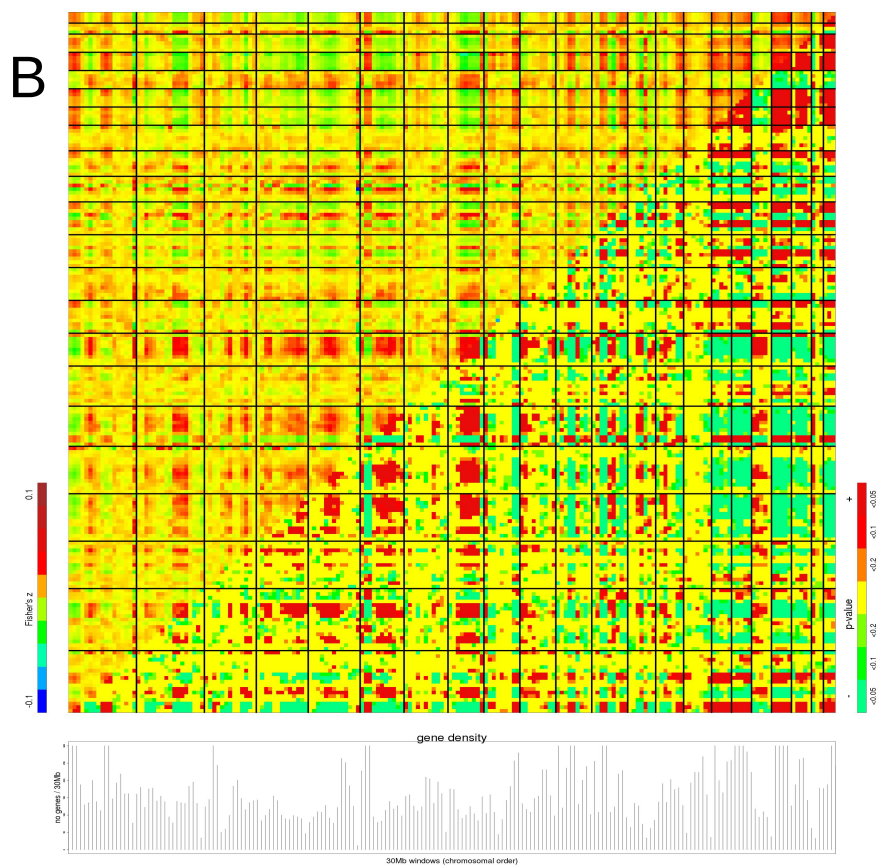

Supplement: Figure S5 — Genome-wide correlation matrix at 30 Mb resolution. See Figure 4 for legend. (A) Adipose Gene Expression from 702 human populations (Table S1). (B) Mouse co-expression,averaged across the mouse expression profiles (Table S1), using the sample size as weight. Correlation heatmaps were generated after reordering mouse genes by their human ortholog locations in the human genome. (2.54 MB PDF) [file pone.0012158.s005.pdf]

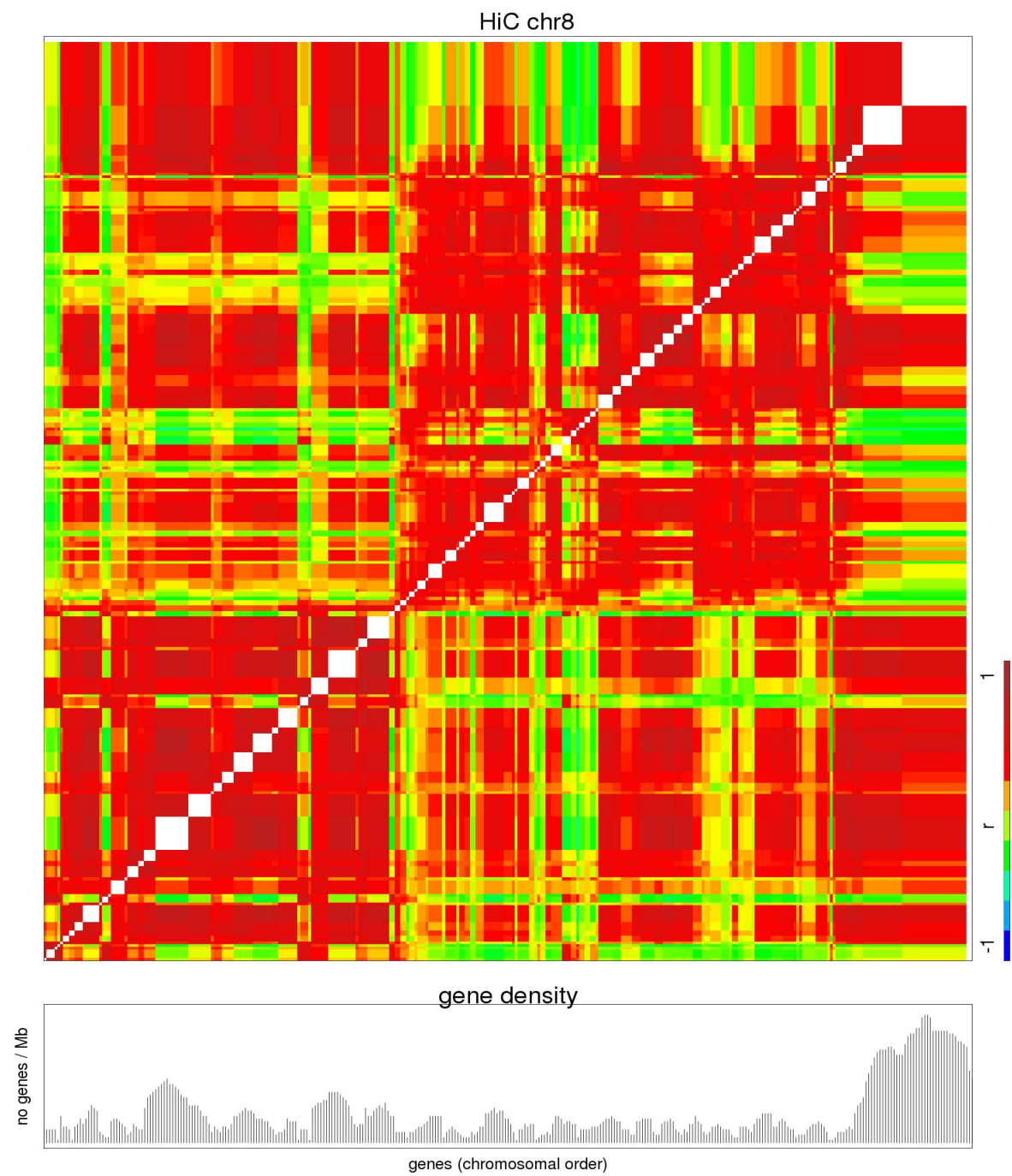

Supplement: Figure S6 — Correlation heatmap of spatial proximity data for human chromosome 8. Spatial proximity information was obtained from Lieberman-Aiden et al. 2009 [15]. The corrrelation ranges from −1 to +1, and greater values indicate greater probability of contact between two genomic domains. Genes whose midpoints falling into the same 1 Mb window are indicated as white. (0.35 MB PDF) [file pone.0012158.s006.pdf]

A

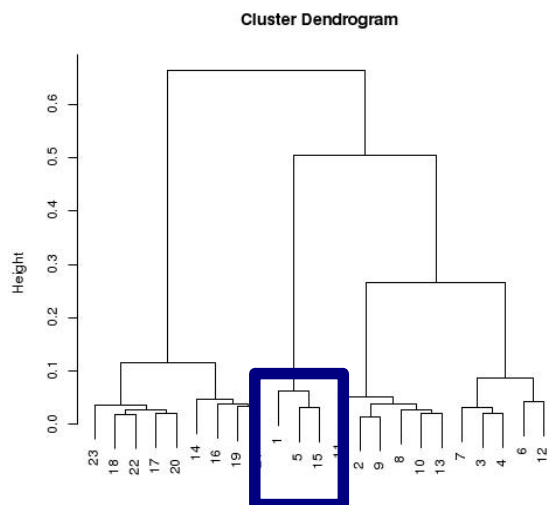

B

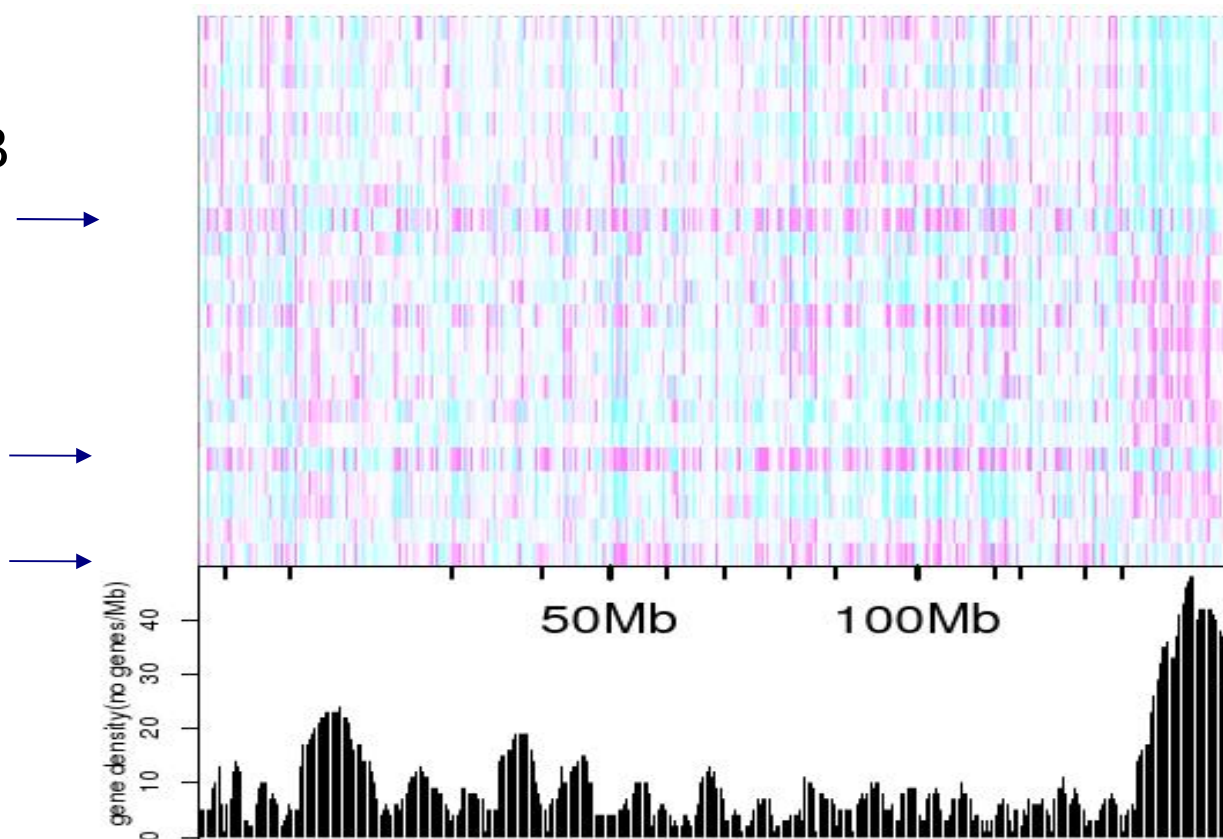

Supplement: Figure S7 — Alzheimer entorhinal cortex gene expression profile. A: Hierarchical clustering of the samples in Alzheimer entorhinal cortex gene expression study [18]. Outliers (boxed blue) were boxed. B: Heatmap showing relative gene expression profiles in diseased (top 10 rows) and normal samples (bottom 13 rows) across genes in chromosome 8 (horizontal,proximal to distal). Magenta and cyan indicate high and low expression. The outliers removed are indicated indicated by arrows. Expression level for each gene were scaled to have a mean of 0, and truncated at −1 and +1 for visualization. (0.08 MB PDF) [file pone.0012158.s007.pdf]

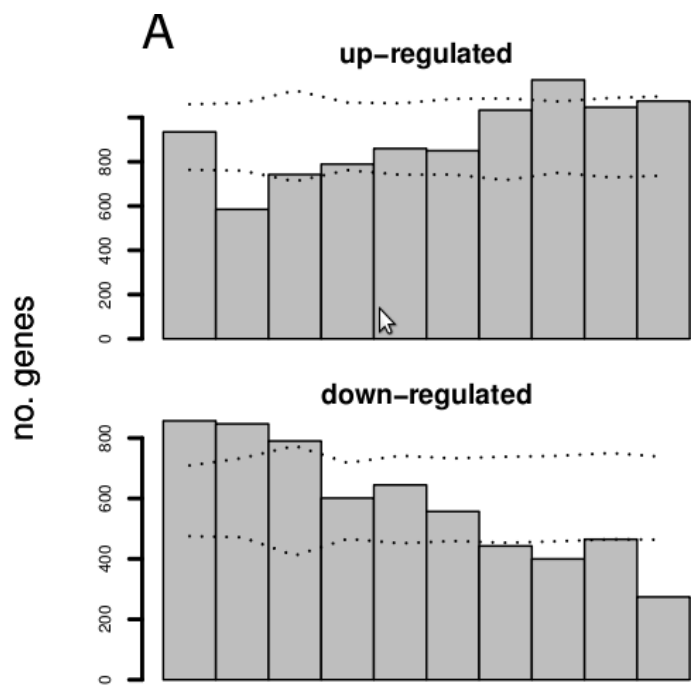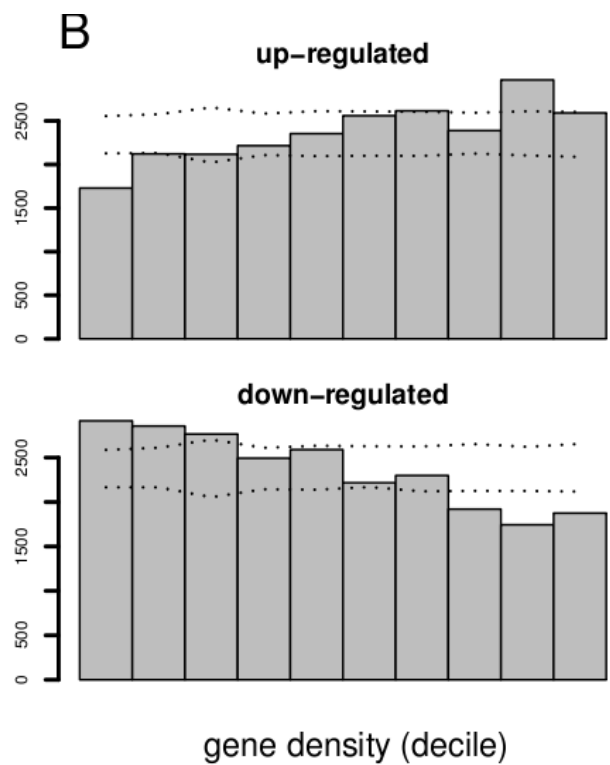

Supplement: Figure S8 — Density-dependent Gene Expression Changes in Diseased Tissues. See Figure 7 for legends. (A) an independent study on hippocampus expression in Alzheimer's disease, corresponding to Figure 7B [19]. (B) an independent study on skin expression in psoriasis patient, corresponding to Figure 7C [21]. (0.05 MB PDF) [file pone.0012158.s008.pdf]
