# Supplementary material for: Coordinated Expression Domains in Mammalian Genomes
Source: PLoS One. 2010 Aug 18;5(8):e12158. doi: 10.1371/journal.pone.0012158 (PMC2923606; doi:10.1371/journal.pone.0012158)
Supplement: Table S1 — Detailed list of gene expression datasets used in the study. (0.03 MB DOC) [file pone.0012158.s009.doc]

| **Species** | **Dataset** | **sample size** | **Description** | **Microarray Platform** | **Primary Perturbation Category** |
| --- | --- | --- | --- | --- | --- |
| Human | (Su et al. 2004) | 73 | Tissue Collection from GNF | Affymetrix U133A | Tissue |
|  | (Göring et al. 2007) | 1240 | Mexican American Lymphocytes | Illumina | Genetic |
|  | (Emilsson et al. 2008) | 701 | Iceland Cohort Adipose | Rosetta/Merck Human 3.0 A1 (Agilent) | Genetic |
|  | (Schadt et al. 2008) | 427 | Liver Biopsy | Rosetta/Merck Human 44k 1.1 (Agilent) | Genetic |
|  | (Lamb et al. 2006) | 2335 | The Connectivity Map: Expression Signature of Small Molecules | Affymetrix U133A | Chemical |
|  | (Liang et al. 2008) | 23 | Hippocampus in Alzheimer's Disease (10) and Normal Controls (13) | Affymetrix U133Plus | Disease |
|  | (Liang et al. 2008) | 23 | Entorhinal Cortex in Alzheimer's Disease (10) and Normal Controls (13) | Affymetrix U133Plus | Disease |
|  | (Blalock et al. 2004) | 31 | Hippocampus in Alzheimer's Disease (22) and Normal Controls (9) | Affymetrix U133Plus | Disease |
|  | (Nair et al. 2009) | 116 | Matched Uninvolved(58) and Lesions(58) from Psoriasis Patients | Affymetrix U133Plus | Disease |
|  | (Reischl et al. 2007) | 26 | Matched Uninvolved(13) and Lesions(13) from Psoriasis Patients | Affymetrix U133A | Disease |
| Mouse | (Su et al. 2004) | 61 | Tissue Collection from GNF (V2) | Affymetrix GNF1m | Tissue |
|  | (Lattin et al. 2008) | 47 | Tissue Collection from GNF (V3) | Affymetrix 430 2.0 | Tissue |
|  | (Chen et al. 2008) | 295 | F2 intercross (BxHAdipose) | Rosetta/Merck 75K oligo array 1 (Agilent) | Genetic |
|  | (Chen et al. 2008) | 311 | F2 intercross (BxHLiver) | Rosetta/Merck 75K oligo array 1 (Agilent) | Genetic |
|  | Unpublished resources | 120 | F2 intercross (BxALiver) | Affymetrix 430 | Genetic |
