# Supplementary material for: Coordinated Expression Domains in Mammalian Genomes
Source: PLoS One. 2010 Aug 18;5(8):e12158. doi: 10.1371/journal.pone.0012158 (PMC2923606; doi:10.1371/journal.pone.0012158)
Supplement: Table S3 — Type III Analysis of variance to dissect density-dependent and spatial proximity-dependent coexpression among intrachromsomal pairs. (0.01 MB DOC) [file pone.0012158.s011.doc]

|  | Df | Sum of Sq | RSS | AIC | F value | Pr(F) |
| --- | --- | --- | --- | --- | --- | --- |
|  |  |  | 44195 | -6764683 |  |  |
| density similarity | 9 | 373 | 44569 | -6749398 | 1708 | 0* |
| spatial proximity | 9 | 234 | 44430 | -6755073 | 1073 | 0* |

* too small to be computed for exact p-value.
