# Supplementary material for: Coordinated Expression Domains in Mammalian Genomes
Source: PLoS One. 2010 Aug 18;5(8):e12158. doi: 10.1371/journal.pone.0012158 (PMC2923606; doi:10.1371/journal.pone.0012158)
Supplement: Reference S1 — (0.02 MB DOC) [file pone.0012158.s012.doc]

# References

Blalock, E. M., Geddes, J. W., Chen, K. C., Porter, N. M., Markesbery, W. R. & Landfield, P. W. 2004. Incipient Alzheimer's disease: microarray correlation analyses reveal major transcriptional and tumor suppressor responses.. *Proc Natl Acad Sci U S A* (101) : 2173-2178

Chen, Y., Zhu, J., Lum, P. Y., Yang, X., Pinto, S., MacNeil, D. J., Zhang, C., Lamb, J., Edwards, S., Sieberts, S. K., Leonardson, A., Castellini, L. W., Wang, S., Champy, M.-F., Zhang, B., Emilsson, V., Doss, S., Ghazalpour, A., Horvath, S., Drake, T. A., Lusis, A. J. & Schadt, E. E. 2008. Variations in DNA elucidate molecular networks that cause disease.. *Nature* (452) : 429-435

Emilsson, V., Thorleifsson, G., Zhang, B., Leonardson, A. S., Zink, F., Zhu, J., Carlson, S., Helgason, A., Walters, G. B., Gunnarsdottir, S., Mouy, M., Steinthorsdottir, V., Eiriksdottir, G. H., Bjornsdottir, G., Reynisdottir, I., Gudbjartsson, D., Helgadottir, A., Jonasdottir, A., Jonasdottir, A., Styrkarsdottir, U., Gretarsdottir, S., Magnusson, K. P., Stefansson, H., Fossdal, R., Kristjansson, K., Gislason, H. G., Stefansson, T., Leifsson, B. G., Thorsteinsdottir, U., Lamb, J. R., Gulcher, J. R., Reitman, M. L., Kong, A., Schadt, E. E. & Stefansson, K. 2008. Genetics of gene expression and its effect on disease.. *Nature* (452) : 423-428

Göring, H. H. H., Curran, J. E., Johnson, M. P., Dyer, T. D., Charlesworth, J., Cole, S. A., Jowett, J. B. M., Abraham, L. J., Rainwater, D. L., Comuzzie, A. G., Mahaney, M. C., Almasy, L., MacCluer, J. W., Kissebah, A. H., Collier, G. R., Moses, E. K. & Blangero, J. 2007. Discovery of expression QTLs using large-scale transcriptional profiling in human lymphocytes.. *Nat Genet* (39) : 1208-1216

Lamb, J., Crawford, E. D., Peck, D., Modell, J. W., Blat, I. C., Wrobel, M. J., Lerner, J., Brunet, J.-P., Subramanian, A., Ross, K. N., Reich, M., Hieronymus, H., Wei, G., Armstrong, S. A., Haggarty, S. J., Clemons, P. A., Wei, R., Carr, S. A., Lander, E. S. & Golub, T. R. 2006. The Connectivity Map: using gene-expression signatures to connect small molecules, genes, and disease.. *Science* (313) : 1929-1935

Lattin, J. E., Schroder, K., Su, A. I., Walker, J. R., Zhang, J., Wiltshire, T., Saijo, K., Glass, C. K., Hume, D. A., Kellie, S. & Sweet, M. J. 2008. Expression analysis of G Protein-Coupled Receptors in mouse macrophages.. *Immunome Res* (4) : 5

Liang, W. S., Dunckley, T., Beach, T. G., Grover, A., Mastroeni, D., Ramsey, K., Caselli, R. J., Kukull, W. A., McKeel, D., Morris, J. C., Hulette, C. M., Schmechel, D., Reiman, E. M., Rogers, J. & Stephan, D. A. 2008. Altered neuronal gene expression in brain regions differentially affected by Alzheimer's disease: a reference data set.. *Physiol Genomics* (33) : 240-256

Nair, R. P., Duffin, K. C., Helms, C., Ding, J., Stuart, P. E., Goldgar, D., Gudjonsson, J. E., Li, Y., Tejasvi, T., Feng, B.-J., Ruether, A., Schreiber, S., Weichenthal, M., Gladman, D., Rahman, P., Schrodi, S. J., Prahalad, S., Guthery, S. L., Fischer, J., Liao, W., Kwok, P.-Y., Menter, A., Lathrop, G. M., Wise, C. A., Begovich, A. B., Voorhees, J. J., Elder, J. T., Krueger, G. G., Bowcock, A. M., Abecasis, G. R. & of Psoriasis, C. A. S. 2009. Genome-wide scan reveals association of psoriasis with IL-23 and NF-kappaB pathways.. *Nat Genet* (41) : 199-204

Reischl, J., Schwenke, S., Beekman, J. M., Mrowietz, U., Stürzebecher, S. & Heubach, J. F. 2007. Increased expression of Wnt5a in psoriatic plaques.. *J Invest Dermatol* (127) : 163-169

Schadt, E. E., Molony, C., Chudin, E., Hao, K., Yang, X., Lum, P. Y., Kasarskis, A., Zhang, B., Wang, S., Suver, C., Zhu, J., Millstein, J., Sieberts, S., Lamb, J., GuhaThakurta, D., Derry, J., Storey, J. D., Avila-Campillo, I., Kruger, M. J., Johnson, J. M., Rohl, C. A., van Nas, A., Mehrabian, M., Drake, T. A., Lusis, A. J., Smith, R. C., Guengerich, F. P., Strom, S. C., Schuetz, E., Rushmore, T. H. & Ulrich, R. 2008. Mapping the genetic architecture of gene expression in human liver.. *PLoS Biol* (6) : e107

Su, A. I., Wiltshire, T., Batalov, S., Lapp, H., Ching, K. A., Block, D., Zhang, J., Soden, R., Hayakawa, M., Kreiman, G., Cooke, M. P., Walker, J. R. & Hogenesch, J. B. 2004. A gene atlas of the mouse and human protein-encoding transcriptomes.. *Proc Natl Acad Sci U S A* (101) : 6062-6067
